# Design and evaluation of dual-function antimicrobial peptides FPON for gram-negative bacteria with membrane disruption and translation inhibition abilities

**DOI:** 10.1128/msphere.00398-25

**Published:** 2025-12-31

**Authors:** Yingqi Tang, Jiye Liu, Wei Zhong, Jianan Tian, Zhixiong Xie, Lipeng Zhong

**Affiliations:** 1 Center for Molecular Diagnosis and Precision Medicine, The First Affiliated Hospital, Jiangxi Medical College, Nanchang University 47861, Nanchang, China; 2 School of Economics and Management, Nanchang University 47861, Nanchang, Jiangxi, China; 3 Hubei Key Laboratory of Cell Homeostasis, College of Life Sciences, Wuhan University 98436, Wuhan, China; 4 The First Clinical Medical College of Nanchang University, Nanchang University 47861, Nanchang, China; 5 The Fourth Clinical Medical College of Nanchang University, Nanchang University 47861, Nanchang, China; 6 Jiangxi Provincial Center for Advanced Diagnostic Technology and Precision Medicine, The First Affiliated Hospital, Jiangxi Medical College, Nanchang University 47861, Nanchang, China; Hackensack Meridian Health Center for Discovery and Innovation, Nutley, New Jersey, USA

**Keywords:** dual function, antimicrobial peptides, LPS, ribosome, multidrug resistant gram-negative bacteria

## Abstract

The situation regarding drug resistance among gram-negative bacteria is becoming increasingly severe. While antimicrobial peptides are an ideal alternative to traditional antibiotics, single-target natural antimicrobial peptides exhibit limitations, including high toxicity and poor permeability. Given the numerous advantages of dual-target peptides for disease treatment, we designed and synthesized the first membrane/ribosome dual-target antimicrobial peptide, FPON, through a functional peptide splicing strategy utilizing FP-CATH and Oncocin as templates. FPON specifically targets gram-negative bacteria and possesses dual functionalities: the ability to disrupt bacterial membrane integrity and the ability to inhibit protein translation. Additionally, FPON exhibited low toxicity and demonstrated significant activity against drug-resistant bacteria *in vitro* and *in vivo*. In conclusion, the results presented in this study provide further evidence that dual-targeted antimicrobial peptides constitute an effective treatment strategy against gram-negative drug-resistant bacteria.

**IMPORTANCE:**

The issue of antibiotic drug resistance in gram-negative bacteria is one of grave urgency. While single-target antimicrobial peptides offer a potential solution to antibiotic resistance, therapeutic applications are constrained by their high toxicity and poor penetration. In this study, FP-CATH and Oncocin were used as templates for functional peptide splicing to develop FPON, a novel antimicrobial peptide. FPON was shown to disrupt bacterial membranes and inhibit protein synthesis, effectively eliminating gram-negative bacteria. Moreover, FPON exhibits low toxicity and has a significant effect against drug-resistant bacteria. Our research demonstrates that a dual-target design offers a promising avenue for addressing drug-resistant infections.

## INTRODUCTION

Antibiotic-resistant gram-negative bacteria present a significant threat to public health (1). Antimicrobial peptides (AMPs) have been proposed as an effective alternative to traditional antibiotics, specifically because they exhibit inherent antibacterial properties and can integrate with the host’s defense system, and the likelihood of resistance is low (2). AMPs can be categorized—based on their mechanisms of action—into those that target membranes and those that target intracellular biomolecules. However, monotarget AMPs have several limitations, including high toxicity and poor permeability. For example, the membrane-disrupting action of melittin has detrimental effects on mammalian cells (3). Similarly, Oncocin, a peptide that targets intracellular ribosomal proteins, has difficulties overcoming the bacterial membrane barrier, with *sbmA* mutations significantly impairing its antibacterial efficacy (4). These problems underscore the urgent need for innovative antibacterial drug design.

The advantages of multitarget drugs in antibacterial therapy are numerous (5). In this study, we demonstrate a novel multitarget strategy for antibacterial treatment by designing a dual-target antibacterial peptide that simultaneously targets the bacterial membrane and ribosome. This approach leverages the synergistic effects of inhibitors of bacterial membranes and ribosomes to increase antibacterial efficacy (6, 7). Our study adopts a pharmacophore fusion strategy based on the binding modes of membrane and ribosome inhibitors, using two templates: FP-CATH (8)—a novel membrane-active peptide discovered in our laboratory—and Oncocin, a previously reported antibacterial peptide (AMP). This approach enabled the design and synthesis of FPON, the first dual-target antibacterial peptide. FPON was demonstrated to exhibit specific antibacterial activity against gram-negative bacteria and to possess dual functionalities: disruption of bacterial membrane integrity and inhibition of protein translation. In summary, the findings presented here provide evidence that FPON effectively targets bacterial lipopolysaccharides (LPS) and ribosomes, exerting antibacterial effects by compromising bacterial membranes and inhibiting protein synthesis.

## MATERIALS AND METHODS

### Bacterial and chemical agents

The strains utilized in this research, namely, *E. coli* BW25113 and its mutant *E. coli* BW25113 Δ*sbmA*, were sourced from the CGSC (Yale University *Escherichia Coli* Genetic Preservation Center), while the clinical isolate was procured from the First Affiliated Hospital of Nanchang University. Additional strains were taken from laboratory reserves. The FPON peptide was synthesized by Wuhan Aoke Dingsheng Biotechnology Co., Ltd, ensuring a purity level exceeding 95%.

### Circular dichroism

For circular dichroism analysis, the methodology was outlined in the referenced study (8). FPON was prepared by dissolving it in PBS buffer supplemented with 25 mM SDS to simulate membrane conditions, achieving a final concentration of 10 μM. Spectral scans were conducted over a wavelength range of 180–260 nm, with each scan repeated three times to ensure accuracy.

### Antimicrobial activity assay

To evaluate antimicrobial efficacy, the protocols established by the Clinical and Laboratory Standards Institute were rigorously adhered to.

### Transmission electron microscopy of FPON damage to bacterial membranes

*E. coli* BW25113 was stored at −80°C, inoculated onto LB plates using the three-zone method, and incubated at 37°C for 12 h. Single colonies were transferred to liquid LB medium and grown at 37°C with shaking at 150 rpm until the OD_600_ reached 0.6–0.8. The bacterial mixture, supplemented with peptide at 5× MIC, was incubated at 37°C for 60 min, then fixed in 0.1 M PBS containing 3% glutaraldehyde and 0.075% rhodamine for 1 h, followed by treatment with 0.075% rhodamine and 1% tetroxopalladium for 2 h. After dehydration through a graded ethanol series, samples were embedded in Spurr resin, cut into thin sections, stained with uranyl acetate and lead citrate, and observed using transmission electron microscopy.

### Time–killing curve

*E. coli* BW25113 Δ*sbmA* was harvested during the logarithmic growth phase (oscillatory culture at 37°C) and diluted with fresh MH liquid medium to achieve a bacterial suspension of 10^6^ CFU/mL. FPON, dissolved in normal saline, was added to the bacterial suspension, resulting in a final concentration of 6.25 μg/mL (2× MIC). The bacterial solution containing the drug was incubated at 37°C for shock culture. At 0, 2, 4, 6, 8, 10, and 12 h, 50 μL of the bacterial solution was diluted 1,000-fold. Subsequently, 50 μL of the diluted bacterial solution was spread on MH solid medium, and the bacterial colonies were counted after overnight culture at 37°C. Saline served as a negative control.

### Hemolytic and cytotoxicity assay of FPON

#### Determination of hemolytic activity

Bovine blood cells were washed three times with normal saline and configured into a 5% erythrocyte suspension. The resulting red blood cell suspension was mixed with FPON dissolved in normal saline at a specified concentration, incubated at 37°C for 1 h, and subsequently centrifuged at 1,000 rpm for 5 min. The supernatant solution was collected, and the absorbance was measured at a wavelength of 540 nm. Normal saline served as a negative control, while 0.2% Triton X-100 was utilized as a positive control. The percentage of hemolysis rate is calculated by the following formula: hemolysis rate % = [*A*_(sample)_ − *A*_(negative control)_] / [*A*_(positive control)_ − A_(negative control)_] × 100%.

#### Determination of cytotoxicity

The cytotoxicity of the drug on mouse Raw264.7 cells was assessed using the CCK-8 method. Briefly, the drug was dissolved in serum-free RPMI 1640 medium and added to a 96-well plate containing mouse Raw264.7 cells (2 × 10^4^ cells/well), with serum-free RPMI 1640 medium without the drug serving as a blank control. Ten microliters of CCK-8 reagent was added to each well. After a 4 h incubation, the absorbance at 450 nm was measured. The results from three independent experiments were averaged, and cell viability was calculated as % = [*A*_(dosed)_ − *A*_(blank)_] / [*A*_0−(dosed)_ − *A*_(blank)_] × 100%. A blank represents the absorbance of the well containing only the medium and CCK solution without cells.

### Measurement of FPON-LPS binding affinity by isothermal titration calorimetry (ITC)

Thermal analysis of ITC was performed using a small-volume ITC calorimeter (MicroCal Auto-iTC200, Malvern, USA). The solution was degassed under vacuum before the experiment. The instrument tubing was cleaned with methanol and washing solution. Two hundred microliters of LPS (0.1 mM, PBS, pH 6.5) was filled into the sample cell of the iTC200 using a syringe, and 50 μL of FPON (0.25 mM, PBS, pH 6.5) was added to titrate the LPS in the sample cell. In fact, the experiment was carried out under continuous stirring at 25℃. The titration was progressive, with 2 μL of FPON injected each time, for a total of 19 injections. The data were analyzed using the software provided by the instrument, and the interaction heat measured after each injection was plotted against time and [peptide]:[LPS] molar ratio. The experiment was repeated three times.

### *In vitro* FPON inhibits translation of bacterial proteins

The inhibitory effect of peptides on the synthesis of GFP in a cell-free system was evaluated using a transcription–translation coupled method based on *E. coli* lysate. Following the instructions provided with the myTXTL Sigma70 Master Mix Kit, the *E. coli* lysate was mixed with 4 μL of the ready-to-use premix and 1 μL of the deGFP plasmid, followed by the addition of 4 μL of reaction buffer. Subsequently, FPON or polymyxin B (1 μL at a concentration of 100 μg/mL) was added, resulting in a total micro-reaction volume of 10 μL, with a final peptide or drug concentration of 10 μg/mL. The impact of the peptides on the *in vitro* transcription–translation was quantified by measuring the expression of the GFP protein. The fluorescence intensity was calculated by measuring the fluorescence signal at *λ*_excitation_ = 460 nm and *λ*_emission_ = 525 nm at the 30 min time point.

### Molecular docking by HDOCK

Molecular docking predicts the binding affinities between protein–ligand, protein–DNA/RNA, and protein–protein/peptide complexes. The HDOCK web server (http://hdock.phys.hust.edu.cn/) is a comprehensive tool for homology search, template-based modeling, structure prediction, macromolecular docking, biological information integration, and job management. It supports amino acid sequences as input and employs a hybrid docking strategy that incorporates experimental data about protein binding sites and small-angle X-ray scattering. HDOCK also supports protein–RNA/DNA docking with an intrinsic scoring function. The structures of ribosomal subunit (PBD ID: 1vy4), LPS, and FPON from the protein database were uploaded to HDOCK, and the DOCK program was run.

### Molecular dynamics simulations

In the present investigation, we employed Amber20 to conduct molecular dynamics simulations on protein–molecule/nucleic acid complexes derived from molecular docking studies. The force field parameters for both proteins and molecules were characterized using the Amber FF19SB and ACPYPE force fields. The three-point transferable intermolecular potential solvent was utilized to solvate the protein–small molecule complexes, ensuring that the nearest distance between protein atoms and the edge of the water box was maintained at a minimum of 1.0 nm (10 Å). Appropriate amounts of Na+/Cl− ions were added along with the solvent to neutralize the system’s charge. To achieve a stable system, energy minimization was performed using the steepest descent algorithm. Following this, the solute was constrained within the constant volume and temperature ensemble, with the system being heated from 0 K to 298.15 K. This heating phase was succeeded by equilibration in the constant pressure and temperature ensemble at 298.15 K and 1 bar pressure. Finally, a 100 ns molecular dynamics simulation was executed on the protein–small molecule complex, with the simulation trajectory saved for later analysis.

### Transcriptome sequencing

Transcriptome sequencing was conducted by Guangzhou Gideo Biotechnology Co., Ltd. Briefly, we incubated *E. coli* BW25113 (OD_600_ ≈ 0.6) with FPON (1× MIC) for 1 h and harvested the bacteria. Total RNA was extracted from each group using TRIzol reagent (Invitrogen). RNA concentration and purity were measured using the NanoDrop 2000 (Thermo Fisher Scientific), and RNA integrity was assessed using the Agilent 2100 Bioanalyzer (RIN ≥ 7.0; Agilent Technologies, Santa Clara, CA, USA). Subsequently, libraries were constructed, and sequencing was performed using the Illumina NovaSeq 6000 platform (Illumina, San Diego, CA, USA). Sequencing data were aligned to the reference genome.

### Bioinformatics analysis

Principal component analysis was employed to assess the reproducibility of the transcriptome sequencing sample data. Differentially expressed genes were filtered using adjusted *P* values of >1.2 as cutoff criteria, and the results were visualized in the form of a volcano plot. Gene Ontology annotation and Kyoto Encyclopedia of Genes and Genomes analyses were performed using the online analysis software package provided by Guangzhou Gideo Biotechnology Co., Ltd. (https://www.omicsmart.com/#/). The results were illustrated as bubble plots, with the significance threshold set at *P* < 0.05.

### *G. mellonella* infection assay

Preparation of bacterial infection samples involved selecting and culturing monoclonal *E. coli* BW25113 Δ*sbmA* in 5 mL of LB broth at a constant temperature of 37°C with shaking at 200 rpm for 12 h. Following this incubation period, the culture was expanded and diluted at a ratio of 1:100. A logarithmic bacterial solution was then prepared, washed with normal saline, and suspended in 1 mL of a solution with a concentration of 1.5 × 10^8^ CFU/mL. A total of four groups were randomly established: *E. coli* BW25113 Δ*sbmA* (treated group), *E. coli* BW25113 Δ*sbmA* + Oncocin, *E. coli* BW25113 Δ*sbmA* + FPON, and NS (saline group). Each group received an injection of 10 μL of the *E. coli* BW25113 Δ*sbmA* bacterial solution (1.5 × 10^8^ CFU/mL). The drug and bacterial solution were mixed thoroughly before being injected into the right hind limb of the larvae. The infected larvae were then incubated in the dark at 37°C without food for up to 7 days. For each group (*n* = 10), survival was monitored post-infection, with observations recorded every 24 h; death was determined when the larvae did not respond to tactile stimulation.

### Statistical analysis

All experiments were repeated three times. Unpaired *t*-tests were used to compare differences between the two groups. For multiple comparisons, one-way analysis of variance followed by Dunnett’s post hoc analysis was used. ns means not statistically significant. Statistical significance is considered with the following *P* values: *P < 0.05, **P < 0.005, ***P < 0.001, and ****P < 0.0001. Using GraphPad Prism 9 software and Kaplan–Meier to calculate *G. mellonella* survival.

## RESULTS

### Synthesis of FPON and evaluation of FPON antimicrobial activity

**Fig 1:**
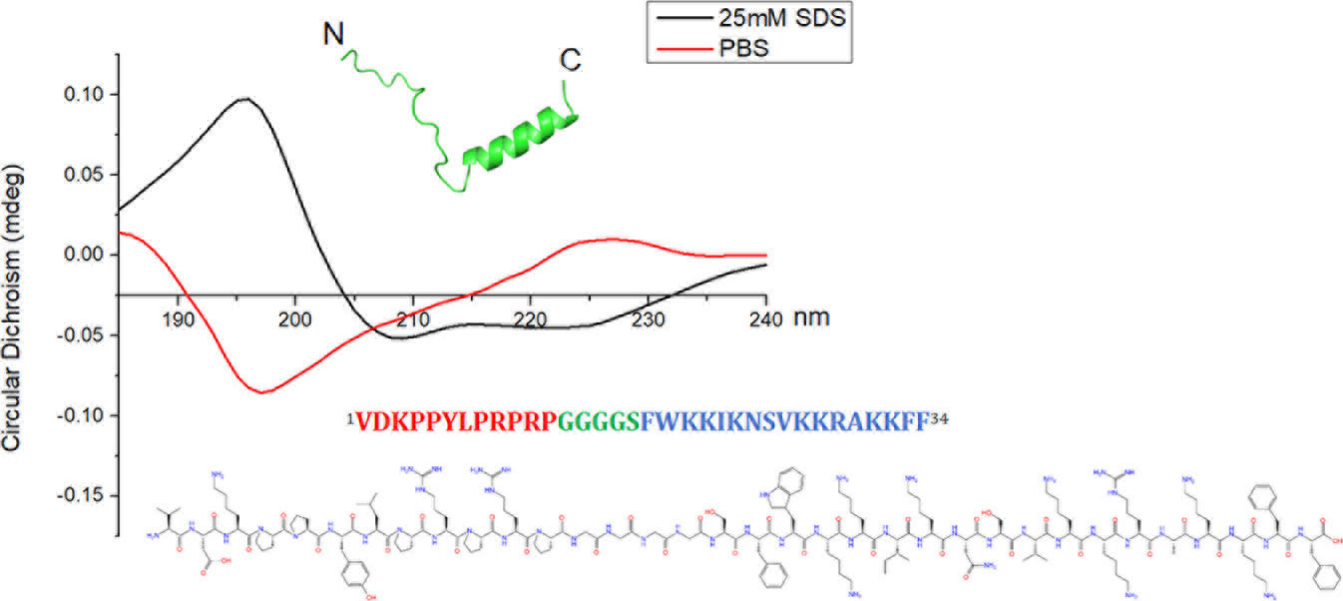
The secondary structure of FPON was analyzed by CD spectroscopy. The solid red line is FPON (10 μM) in PBS buffer, and the solid black line is FPON in 25 mM SDS (the membrane simulation condition).

FPON (^1^VDKPPYLPRPRPGGGGSFWKKIKNSVKKRAKKFF^34^, Mw = 3915.6 , pI = 11.7) is a 34-amino acid-long dual-target antibacterial peptide (Fig. 1). To create FPON, an active peptide derived from Oncocin (underlined segment) was joined to an active peptide derived from FP-CATH (italicized segment) using a flexible linker (boldfaced segment). Under membrane simulation conditions (25 mM SDS), FPON was observed to maintain an α-helical structure. To investigate FPON activity, the antimicrobial activities and profiles of FPON, FP-CATH17a (^1^FWKKIKNSVKKRAKKFF^17^), and Oncocin were evaluated in parallel. Notably, FPON demonstrated specific activity against gram-negative bacteria and maintained efficacy against *Escherichia coli* BW25113 Δ*sbmA* (see Table 1).

**TABLE 1:** Antimicrobial activity of three types of peptides^*a*^

Bacterial strain	MIC (μg/mL)		
	FPON	FP-CATH17a	Oncocin
Gram-negative bacteria			
*E. coli* BW25113	3.12	12.5	6.25
*E. coli* BW25113 Δ*sbmA*	3.12	12.5	100
*Klebsiella pneumoniae* ATCC 700603	6.25	50	25
*Pseudomonas aeruginosa* ATCC 27853	6.25	50	25
*Acinetobacter baumannii* ATCC 19606	6.25	50	25
Gram-positive bacteria			
*Staphylococcus aureus* ATCC 29213	>400	>400	>400
*S. aureus* MRSA	>400	>400	>400

^*a*^ MIC, the minimum inhibitory concentration. The MIC values represent the medians from three independent replicate experiments performed in triplicate.

**Fig 2:**
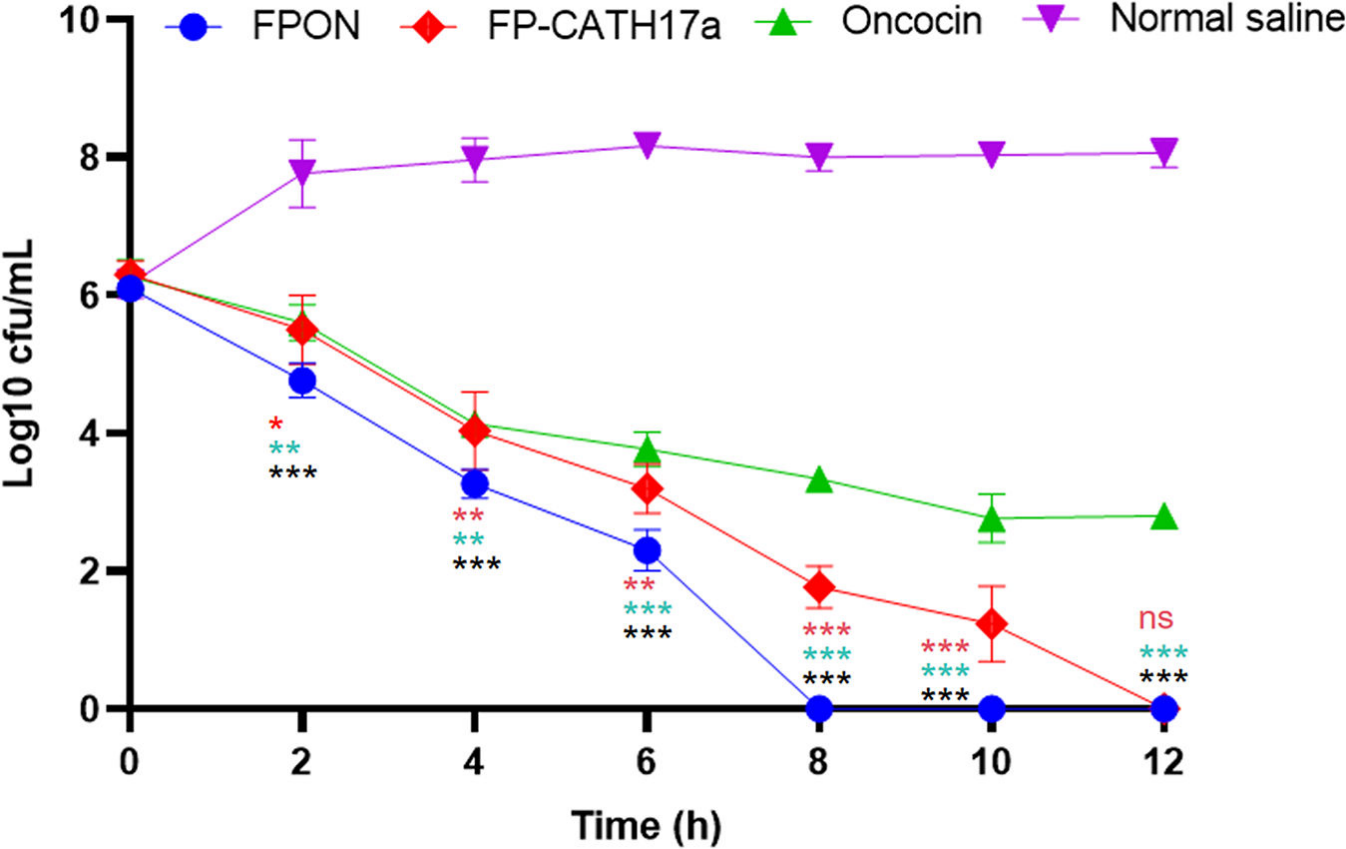
Time-killing curves of three types of peptides. The values represent the mean ± SD (*n* = 3) expressed as log10 CFU/mL. Three different peptides (FPON, Oncocin, and FP-CATH17a) were added to the culture medium of the initial bacterial culture. Normal saline was used as the negative control. CFU data of the FPON group were compared to normal saline, Oncocin, and FP-CATH17a group at 2, 4, 6, 8, 10, and 12 h time points using one-way analysis of variance, followed by Dunnett’s test. Significant differences between the treated cells and the control are indicated by asterisks (ns, not significant; *P < 0.05; **P < 0.005; ***P < 0.001 ). represents FPON vs FP-CATH17a; * represents FPON vs Oncocin; * represents FPON vs normal saline.

For efficient sterilization, a ≥3 log reduction (99.9% kill rate) is the generally accepted target. Hence, a drug is considered to exhibit a bactericidal effect on bacteria at concentrations greater than (or equal to) the concentration that elicits a 99.9% kill rate. In the present study, time–kill kinetics assays revealed that 2× minimum inhibitory concentrations (MICs) of FPON rapidly eradicated *E. coli* BW25113 Δ*sbmA* (Fig. 2). While a deceleration in the bactericidal rate was observed after 2 h treatment, near-complete pathogen elimination was obtained within 8 h.

**Fig 3:**
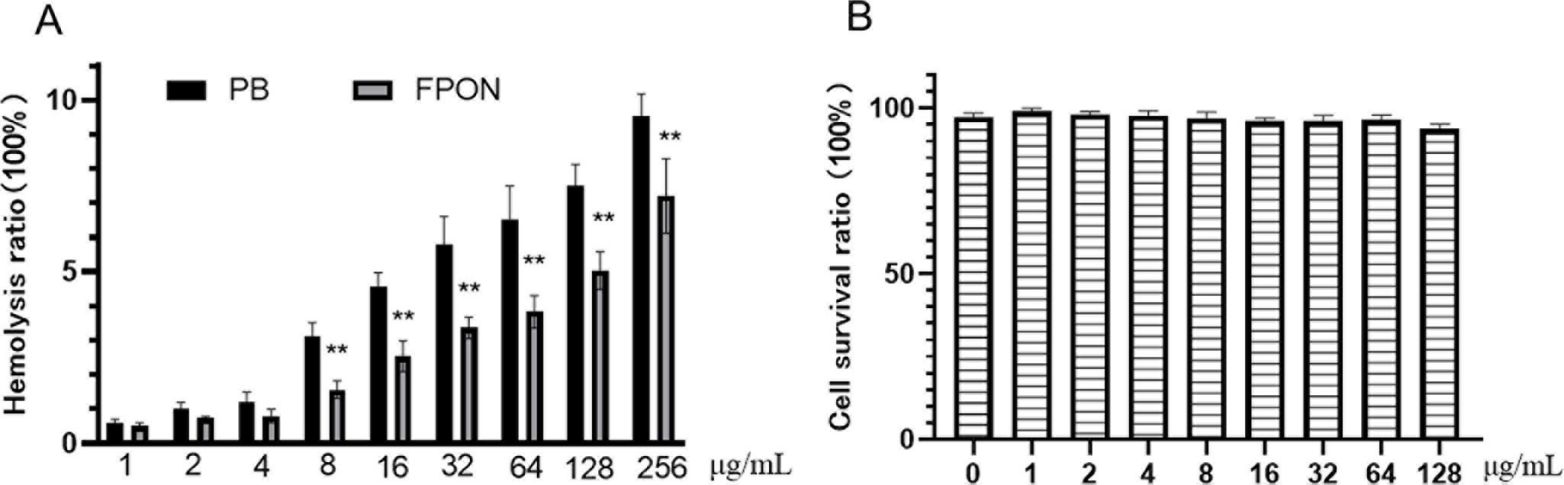
Hemolytic activity of FPON on erythrocytes and cellular toxicity. (**A**) FPON peptide toxicity on bovine erythrocytes. ** represents a statistically significant difference between the FPON group vs the polymyxin B group; **P < 0.005. (**B**) Cytotoxicity of FPON peptide to cells. The results are reported as the means ± SD from three independent experiments.

Additionally, the biocompatibility of FPON was found to be favorable. In a hemolytic activity assay (Fig. 3A), the activity of FPON was only 7.7% ± 1.08% at the highest concentration tested. Importantly, hemolytic activity was significantly lower than bactericidal activity at effective bactericidal concentrations. Similarly, FPON demonstrated low activity in a cytotoxicity assay (Fig. 3B), with a cell survival rate of 93.67% ± 1.53% at a concentration of 128 μg/mL.

### FPON disrupts the cell membrane of gram-negative bacteria by targeting LPS

**Fig 4:**
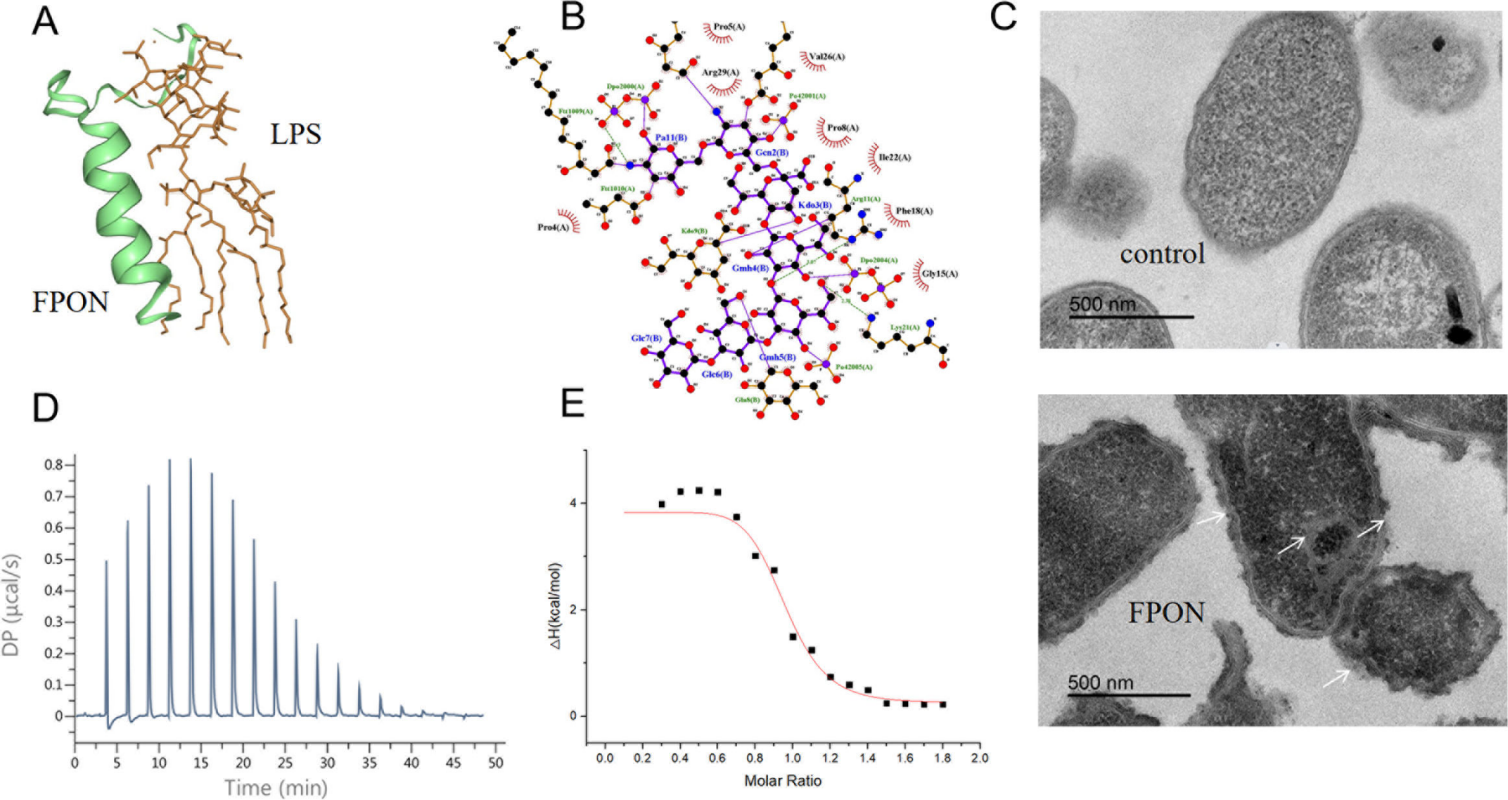
Illustration of the impact of FPON on the integrity of the *E. coli* BW25113 bacterial membrane. (**A**) A three-dimensional spatial diagram depicting the interaction between FPON and LPS. (**B**) A two-dimensional spatial diagram showing the interaction of FPON with LPS. (**C**) Transmission electron microscopy images revealing the effects of physiological saline (control) and FPON on the surface and microstructure of the bacteria. (**D**) A graph depicting the trend of heat change over time. (**E**) An affinity fitting curve.

The molecular docking results (Fig. 4A and B) indicate that polar and charged amino acids in FPON, including proline and arginine, are involved in interactions with LPS. Isothermal titration calorimetry (ITC) results (Fig. 4D and E) reveal that the interaction between FPON and LPS induces an exothermic reaction, typically indicative of conformational changes and hydrophobic interactions. The dissociation constant (*K*_d_) for FPON binding to LPS was 0.45 ± 0.11 μM. The titrations for LPS and the peptides indicated that in the initial 16 drops of reaction titration step, there is the endothermic reaction process, followed by the not obvious exothermic reaction process. The binding saturation was observed at the molar ratio for FPON (peptide:LPS = 1.8) after 45 min with negative enthalpy changes (*ΔH*). Additionally, FPON demonstrated a significant positive charge binding advantage, suggesting that the interaction with LPS is mediated by both electrostatic and hydrophobic forces. Transmission electron microscopy revealed pronounced changes in *E. coli* BW25113 membrane structure and intracellular organization following FPON treatment at 5× MIC for 1 h (Fig. 4C). Notably, the surfaces and membranes of *E. coli* BW25113 cells treated with FPON (Fig. 4C) exhibited significantly more irregularities compared to the control group (Fig. 4C), with increased aggregation and condensation observed intracellularly.

### FPON inhibits bacterial protein translation *in vitro*

**Fig 5:**
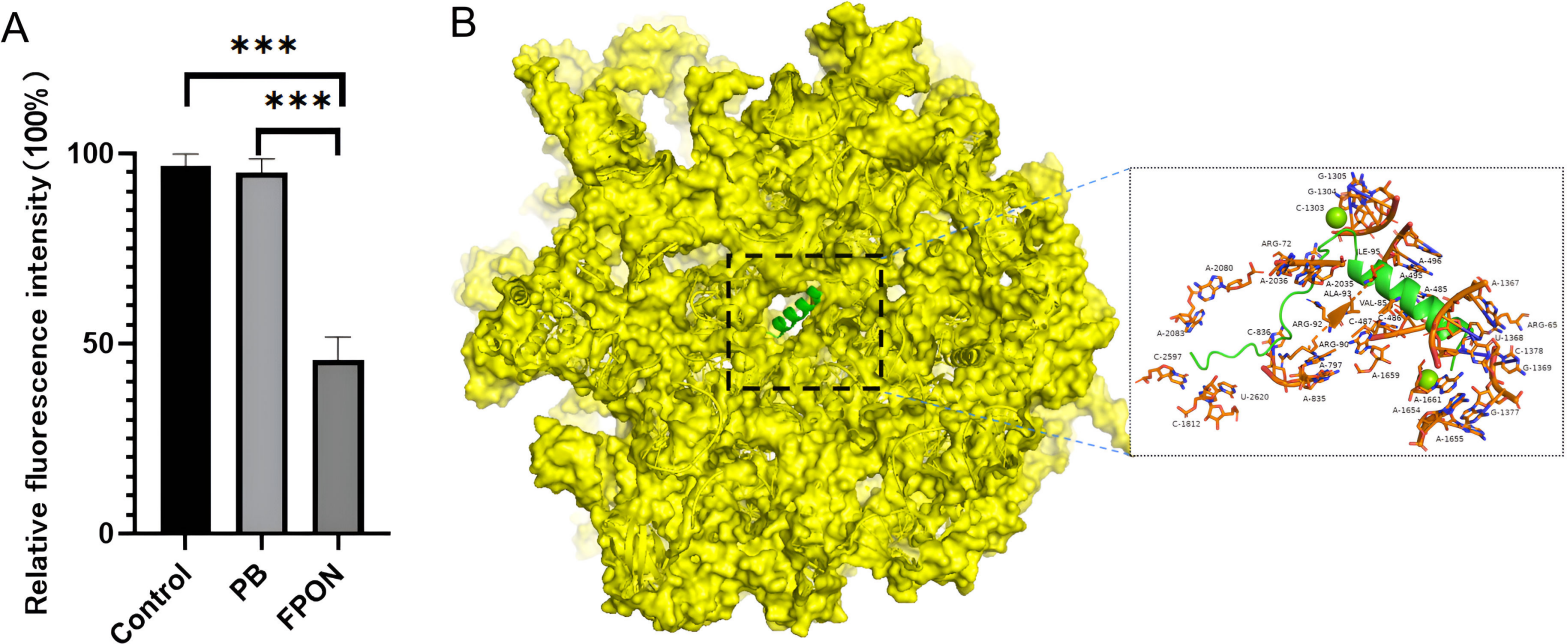
Illustration of the inhibitory effect of FPON on ribosomes. (**A**) *In vitro* expression of GFP was evaluated by mixing the peptide with *E. coli* 70S ribosome and GFP. Control: physiological saline. PB, polymyxin B. Statistical difference: ***P < 0.001. (**B**) Molecular docking prediction of the FPON interaction with the ribosome, where the yellow area represents the bacterial 70S ribosome (PDB ID: 1vy4), and the green area represents FPON.

To assess its functional impact on protein translation, the fluorescence intensity of GFP expression was monitored in a cell-free assay after FPON treatment. For comparison, polymyxin B was used as a negative control. The final concentration of each peptide was set at 10 µg/mL. As shown in Fig. 5A, GFP fluorescence intensity was measured within 30 min of each treatment. The results reveal that FPON inhibition of fluorescence activity was significantly stronger (compared to polymyxin B inhibition).

The results of peptide–ribosome molecular docking studies are shown in Fig. 5B. FPON may interact with amino acids such as Arg, Ile, and Val on ribosomal subunits, as well as nucleic acid molecules such as A, U, G, and C. They suggest that FPON may inhibit the normal functioning of bacterial protein translation by occupying the protein translation channel deep within the ribosomal subunit.

### Molecular dynamics simulations of the FPON–LPS and FPON–ribosomal 70S molecular docking complexes

Molecular dynamics simulations can provide valuable insights into structural changes at the molecular level, capture the time scale of conformational changes, and assess the stability of target complexes. During biomolecular simulations, various metrics are known to provide valuable information. The root mean square deviation (RMSD) is a key metric used to evaluate the accuracy and stability of simulated structures. The radius of gyration (Rg) serves as an important index for evaluating the stability and folding state of proteins. The solvent-accessible surface area (SASA) reveals the function, folding state, and molecular interactions of a protein. By monitoring temporal changes in the number of hydrogen bonds, researchers can gain a deeper understanding of the dynamics of ligand binding to a protein. The root mean square fluctuation (RMSF) quantifies the fluctuations of atoms within a protein throughout the simulation. Lastly, integration of a three-dimensional landscape map of the binding free energy allows for an assessment of changes in the binding free energy of small molecules to proteins.

**Fig 6:**
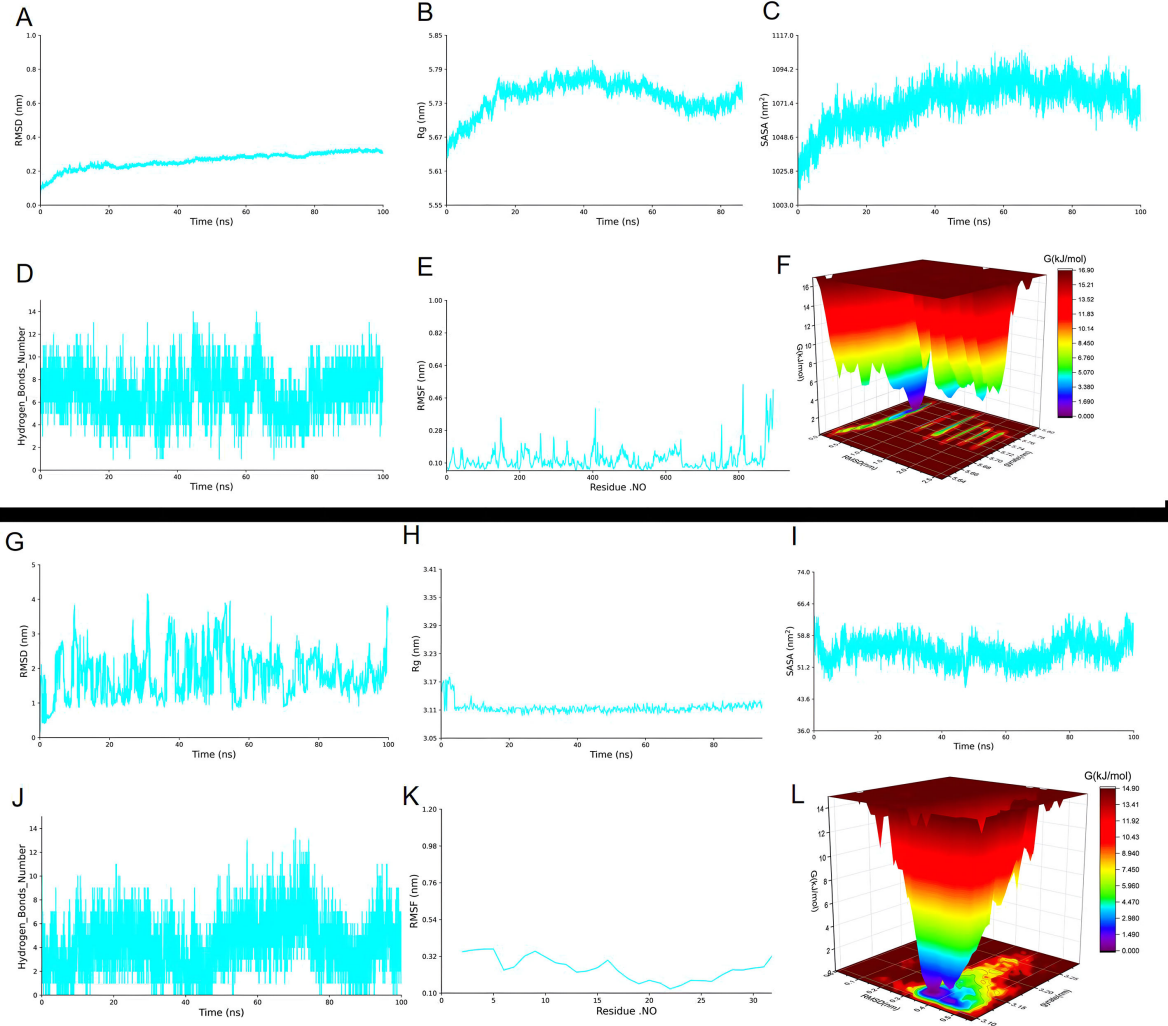
The 100 ns molecular dynamics results for two types of ligand–target complexes. The first section details the findings for FPON–LPS complexes, including (**A**) root mean square deviation (RMSD), (**B**) radius of gyration (Rg), (**C**) solvent-accessible surface area (SASA), (**D**) number of hydrogen bonds, (**E**) root mean square fluctuation (RMSF), and (**F**) a combined 3D landscape map of binding free energy. The second section outlines the results for FPON–ribosome complexes, featuring (**G**) RSMD, (**H**) Rg, (**I**) SASA, (**J**) number of hydrogen bonds, (**K**) RMSF, and (**L**) a combined 3D landscape map of binding free energy.

Here, molecular dynamics simulations were employed to evaluate the stability of FPON–LPS and FPON–ribosome complexes. As shown in Fig. 6A (for the FPON–LPS complex), the RMSD curve of the ligand exhibited minimal fluctuations between 0 and 100 ns, with its value remaining below 0.3 nm. Specifically, RMSD values increased from 0.12 to 0.23 nm during the 0–20 ns interval and fluctuated between 0.23 and 0.3 nm during the 21–100 ns period. The Rg value of the complex increased from 5.64 to 5.8 nm during the 0–40 ns interval (Fig. 6B), indicating that the structure of the complex became more “loose.” Over the course of the 41–100 ns simulation, the Rg value of the complex subsequently decreased from 5.8 to 5.7 nm, suggesting that the structure of the complex became more compact during this stage. During the last 20 ns, the Rg value slightly increased, suggesting that the system again became loose. As shown in Fig. 6C, SASA initially increased over the first 67 ns, indicating a loosening of the system’s structure, before subsequently fluctuating between 1,048 and 1,080 nm². Additionally, the number of hydrogen bonds between the complex varied from 1 to 13 (Fig. 6D), although 4–10 hydrogen bonds were maintained for the majority of this time. From Fig. 6E, it is evident that the RMSF of each residue in the complex remained relatively low overall, suggesting that the flexibility of most residues was maintained within a narrow range. This observation, in conjunction with the peptide’s RMSD change throughout the simulation, indicates a trend toward dynamic stability for the entire peptide. As shown in Fig. 6F, a low-energy conformation was predominant throughout the simulation, suggesting that the peptide–LPS molecule interaction was stable.

As shown in Fig. 6G (for the FPON–ribosome complex), the RMSD curve for the peptide–ribosome complex fluctuates around 2.0 nm during the 0–100 ns time frame (within a 2.0 nm range), which suggests that the complex maintained dynamic stability. Figure. 6H reveals significant changes in the Rg of the complex during the first 0–4 ns, followed by fluctuations around 3.12 nm from 5 to 100 ns (with a minor variation of ~0.03 nm), indicating relative stability of the complex system. In Fig. 6I, the SASA of the complex during the 0–100 ns period fluctuated around 55 nm² (with an amplitude of approximately 6 nm²), suggesting that the overall architecture was maintained (notwithstanding dynamically stable changes). The number of hydrogen bonds between the complex molecules varied from 0 to 13 (Fig. 6J), with two to eight hydrogen bonds maintained for the majority of the simulation. This reflects the contribution of these hydrogen bonds to molecular protein binding. Lastly, the RMSF values for each residue over the course of the simulation indicate that, with the exception of the first and last residues, there was minimal change in the flexibility of each amino acid residue (Fig. 6K). A dynamic curve analysis of the RMSD indicates that the complex exhibited overall dynamic stability throughout the simulation. As shown in Fig. 6L, the free energy landscape of the protein reveals that the dominant conformational state was located within an RMSD range of 0.2–0.58 nm and an Rg range of 3.09–3.25 nm, a region characterized by several energy-stable wells. During the simulation, this low-energy conformation prevailed as the primary conformation, suggesting stable binding of the small molecules to the protein during this phase. Consequently, the entire target ligand–protein complex system demonstrated considerable stability throughout the 0–100 ns simulation period.

### *In vivo* activity assay experiments in a *Galleria mellonella* model

**Fig 7:**
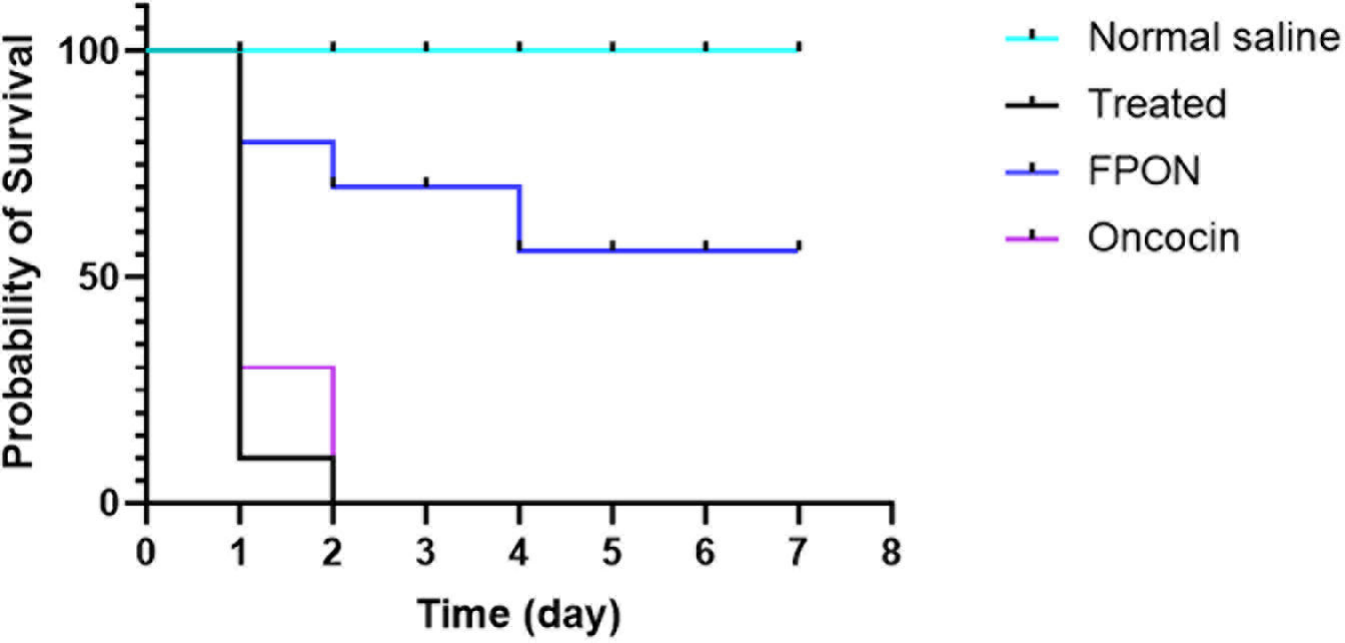
Protective effects of FPON on *G. mellonella* larvae. *E. coli* BW25113 Δ*sbmA* was used as the test strain, and each group consisted of 10 *G*. *mellonella* larvae, which were examined for survival within 7 days. *P* = 0.0017, FPON vs treated; *P* = 0.0053, FPON vs Oncocin.

FPON demonstrated significant protective effects against *E. coli* BW25113 Δ*sbmA* infection in *G. mellonella* (*n* = 10；Fig. 7). No fatalities were observed in *G. mellonella* injected with saline solution over a 7-day period. Conversely, all *G. mellonella* injected with *E. coli* BW25113 Δ*sbmA* succumbed within 1 day. In control experiments, all *G. mellonella* injected with Oncocin + *E. coli* BW25113 Δ*sbmA* died within 2 days. In comparison, *G. mellonell*a injected with FPON + *E. coli* BW25113 Δ*sbmA* survived until the seventh day, demonstrating an overall protective rate of 60%.

### Transcriptomic-level study on the mechanism of FPON action

**Fig 8:**
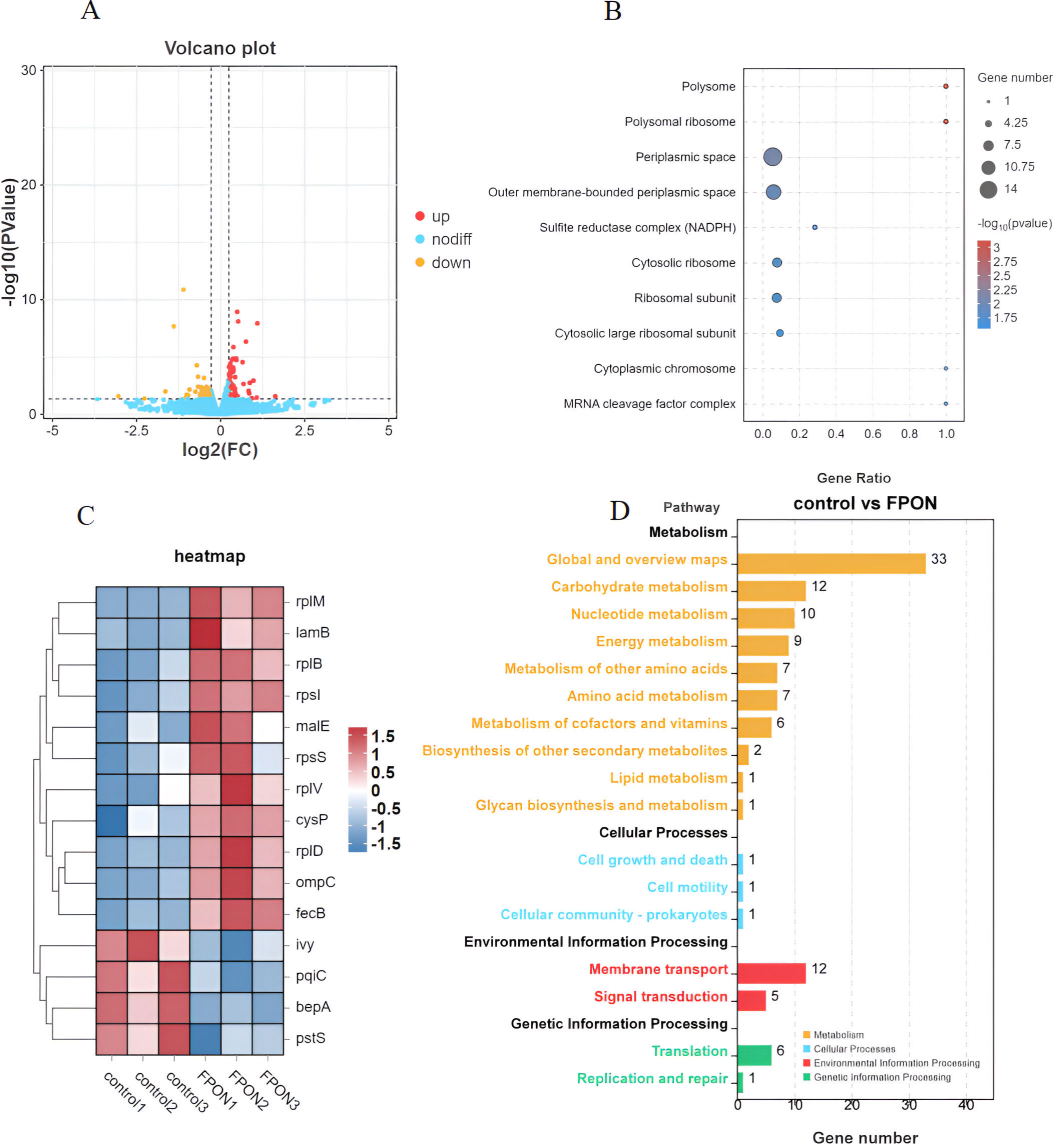
The transcriptomic-level exploration of the FPON mechanism. (**A**) The impact of FPON on the transcriptome of *E. coli* BW25113 is presented. Differentially expressed genes were identified using the criteria |log_2_ FC| ≥ 1.2 and *P* < 0.05, and these genes were visualized in a volcano plot. (**B**) The bacterial signaling pathways enriched through Gene Ontology analysis are depicted. (**C**) A heat map showcasing representative differentially expressed genes is provided. (**D**) The bacterial signaling pathways enriched by Kyoto Encyclopedia of Genes and Genomes analysis of the differential genes are also illustrated.

The effects of FPON on the transcriptional activity of *E. coli* BW25113A were revealed in a preliminary transcriptomic investigation. After incubation of *E. coli* BW25113 (optical density at 600 nm [OD_600_] ≈ 0.6) with FPON (1× MIC) for 1 h and subsequent harvesting of the bacteria, transcriptomic analysis revealed that 46 genes were upregulated and 55 genes were downregulated (Fig. 8A). Differential gene analysis demonstrated that the enriched genes were primarily associated with bacterial ribosomal proteins (*rplM*, *rplV*, *rplD*, *rplB*, *rpsS*, and *rpsI*) (Fig. 8C), as well as signaling pathways related to bacterial outer membrane protein (*ompC*) and membrane protein maturation-related periplasmic proteinase (*bepA*) (Fig. 8B and D).

## DISCUSSION

By 2050, it is estimated that over 10 million people worldwide will die annually from antibiotic-resistant infections (9). Among the antibiotic-resistant bacteria identified, gram-negative bacteria are known to pose a particular challenge. In fact, approximately 70% of clinically isolated resistant strains are gram-negative bacteria, with *E. coli* strains alone accounting for about 20% (10). This alarming trend has prompted scientists to explore new antibacterial strategies, leading to the development of AMPs as potential alternatives to conventional antibiotics.

AMPs are integral to the host’s natural defense system, and these small peptides—from 10 to 100 amino acid residues—exhibit significant antibacterial activity (11). However, natural AMPs have inherent limitations, including toxicity and poor permeability. In particular, when targeting gram-negative bacteria, Oncocin (a proline-rich antimicrobial peptide) generally enters the cytoplasm via specific transporter-mediated mechanisms, especially those involving *sbmA*, to exert their effects. This reliance on specific membrane proteins facilitates the development of bacterial resistance through mutations in these transporters. A study investigating the mutation timeline of evolutionary resistance genes to colistin in Enterobacteriaceae indicates that mutations occur in genes such as *sbmA*, *fadR*, *acpP*, *qseC*, *mgrB*, *phoP*, and *arnC* and that the affected genes are involved in peptide transport, fatty acid biosynthesis, biofilm formation, and regulation of LPS modification (12).

To address these challenges, researchers have initiated hybridization studies—the joining together of two or more AMPs—to exploit their distinct advantages, thereby creating novel antibacterial agents with dual or multifunctional properties. This strategy can be used to enhance the activity and specificity of the peptides and also to mitigate their toxicity. For instance, the hybrid peptide Lf-KR, which is formed by combining LfcinB6 and KR-12, demonstrates high antibacterial, anti-inflammatory, and antibiofilm activities (13). Furthermore, the combination of Aurein with other membrane-penetrating peptides has proven effective in suppressing multiple drug-resistant pathogenic bacterial strains. Spiral peptides, characterized by their unique oblique membrane insertion mechanism and flexible conformation, have emerged as another research hotspot (14). A fusion of NCR247C with truncated Mastoparan generated the chimeric peptide NCR247, which demonstrates significantly enhanced bactericidal efficacy and an altered antibacterial spectrum (15). Similarly, the novel antibacterial peptide, MAA-41, designed by combining LL-37 and BMAP-28, exhibits not only lower toxicity but also antibiofilm properties (16). Moreover, MAA-41 demonstrates synergistic effects when used in conjunction with conventional antibiotics.

### Conclusions

**Fig 9:**
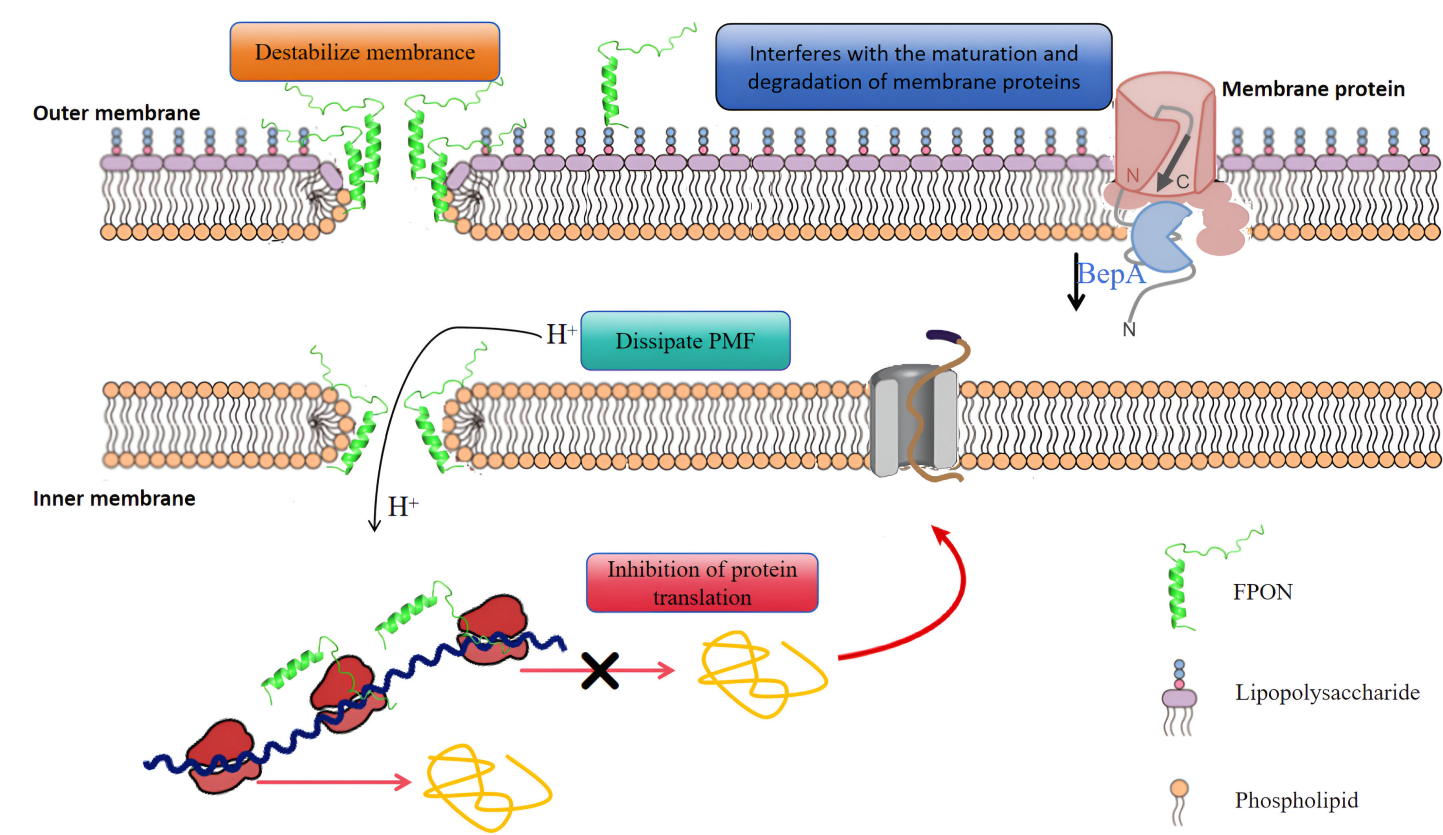
The potential dual-target antimicrobial mechanisms of FPON. In concert, FPON activities impair normal bacterial metabolism and hinder the repair of bacterial membrane damage, ultimately leading to bacterial death from the combined pressures of compromised membrane integrity and stalled protein translation.

Despite the limited research on dual-target antibacterial peptides, we demonstrate in this study that FPON is a promising candidate with potent antibacterial effects against gram-negative bacteria and high biocompatibility (low hemolytic activity and low cytotoxicity). FPON achieves its dual mechanism of action through the synergistic activity of several key amino acids. The proposed antibacterial mechanism of FPON (shown in Fig. 9) suggests that it exerts a dual-function antibacterial effect through multiple key amino acids. FPON antibacterial activity first disrupts the integrity of the bacterial membrane, facilitating accumulation of the peptide within the bacterial cytoplasm, where it simultaneously inhibits the bacterial protein translation process. Specifically, it disrupts the integrity of bacterial cell membranes, facilitating the accumulation of peptides within the cells, where they simultaneously interfere with the bacterial protein translation process, thereby disrupting the pathogen’s normal metabolic pathways. Ultimately, the dual pressures of compromised membrane integrity and impaired protein synthesis lead to pathogen death. This research offers valuable insights for the development of novel, highly efficient, and low-toxicity dual-function antibacterial agents and paves the way for addressing the increasingly serious issue of bacterial resistance.
